# Sudden cardiac death in children and adolescents (excluding Sudden Infant Death Syndrome)

**DOI:** 10.4103/0974-2069.74035

**Published:** 2010

**Authors:** Kelly K Gajewski, J Philip Saul

**Affiliations:** Department of Pediatrics, Louisiana State University School of Medicine, New Orleans, Louisiana, USA; 1 Department of Pediatrics, Medical University of South Carolina, Charleston, South Carolina, USA

**Keywords:** Children, hypertrophic cardiomyopathy, long QT syndrome, sudden cardiac death

## Abstract

Sudden death in the young is rare. About 25% of cases occur during sports. Most young people with sudden cardiac death (SCD) have underlying heart disease, with hypertrophic cardiomyopathy and coronary artery anomalies being commonest in most series. Arrhythmogenic right ventricular dysplasia and long QT syndrome are the most common primary arrhythmic causes of SCD. It is estimated that early cardiopulmonary resuscitation and widespread availability of automatic external defibrillators could prevent about a quarter of pediatric sudden deaths.

## INTRODUCTION

Excluding the Sudden Infant Death Syndrome (SIDS), which has an incidence around 1–1.5/1000 infants, sudden death in a young person is a rare event. Yet, it has a devastating impact when it occurs precisely because it is so unexpected. Sudden cardiac death (SCD) is defined as death that is abrupt, unexpected, and due to a cardiovascular cause. It is generally recognized as death that occurs within 1 hour from the onset of cardiovascular symptoms. However, in young people, it typically occurs within a few minutes of symptom onset. When resuscitation restores spontaneous circulation, it is referred to as “aborted sudden death.” The estimated incidence of pediatric SCD ranges from 0.6 to 6.2 per 100,000 children in the United States.[1–5] Approximately 20–25% of the deaths occur during sports.[6] In patients with congenital heart disease, the numbers are higher and are estimated at 100 deaths per 100,000 patients.[7] In comparison, adults experience SCD at a rate of 400,000 per year (or 135/100,000 population). Since most sudden deaths have a cardiovascular cause, it is theoretically possible to identify the patients at risk prior to the event and prevent it.

## ETIOLOGIES OF SCD IN THE YOUNG

There are several known causes of sudden death in young people [Table 1]. These etiologies can be divided into two categories: arrhythmic and non-arrhythmic. The majority of SCD in young people occurs due to arrhythmic causes. These usually result in an abrupt loss of consciousness, with or without a sensation of palpitations. The arrhythmia may be proven or presumed. Non-arrhythmic etiologies result in circulatory collapse in the setting of an organized rhythm. Examples of the latter include congestive heart failure, embolic phenomena, or aneurysm ruptures. A variety of identifiable conditions have been associated with SCD in the young.

**Table 1:** Causes of sudden death[8]

Causes	Relative incidence (%)
Hypertrophic cardiomyopathy	36
Increased cardiac mass	10
Coronary artery anomalies	24
Marfan’s syndrome	6
Congenital heart disease	5
Myocarditis	3
Dilated cardiomyopathy	3
Arrhythmogenic right ventricular dysplasia	3
Ischemic heart disease	2
Commotio cordis	<1

## HIGH RISK POPULATIONS

### Hypertrophic cardiomyopathy

Hypertrophic cardiomyopathy (HCM) is the most common cause of SCD in the United States in people 30 years old or younger.[8,9] The disease prevalence has been estimated as high as 1 per 500 in young adults.[10,11] It is typically non-obstructive and presents in mid to late adolescence. HCM is often clinically silent, but the ECG typically may show left ventricular hypertrophy or T-wave abnormalities. The diagnosis traditionally has been best confirmed by echocardiography. However, carriers of an HCM genetic mutation may have little or no hypertrophy, especially earlier in life. Associated sudden death is often exertional and is usually secondary to malignant ventricular arrhythmias. HCM is an autosomal dominant congenital disorder typically characterized by asymmetric septal hypertrophy and marked disarray of ventricular muscle fibers, which contribute to the risk of arrhythmias even in patients with minimal hypertrophy and no evident left ventricular outflow tract obstruction. HCM has been found to be caused by genetic abnormalities involving primarily sarcomeric contractile proteins (such as b-myosin and troponin T). However, there is marked genetic and phenotypic heterogeneity. To date, over 20 HCM-susceptibility genes have been identified, and penetrance has been estimated at 30–80%, based on the particular mutation. The Heart Failure Society of America’s 2009 practice guidelines support genetic testing of the one most clearly affected person in a family to facilitate family screening and management (level of evidence A). Genetic testing even in clinically apparent disease may have prognostic significance.[12,13,14] Risk factors for sudden death in patients with HCM include septal thickness ≥30 mm, family history of sudden death, non-sustained VT, syncope, and hypotensive response to exercise. Restriction from most competitive athletics is recommended in patients with HCM.[15,16]

### Dilated cardiomyopathy

Although less common than HCM, dilated cardiomyopathy (DCM) is also a known cardiac risk factor for SCD. DCM is characterized by cardiac dilation and decreased systolic function. It can be acquired from ischemic injury, myocarditis or toxins, or it can be inherited, usually as an autosomal dominant trait, with variable penetrance. There are currently more than 20 different gene mutations identified that cause DCM. The genes responsible encode diverse myocyte proteins including those of the sarcomere, cytoskeleton, nucleus, sarcoplasmic reticulum, and the cell membrane. It is estimated that 20–50% of “idiopathic” DCM has a genetic basis. Although the disease is progressive and often clinically silent in childhood, SCD can occur prior to the development of heart failure and symptoms. Genetic testing may help to identify those at risk for sudden death prior to heart failure. Lamin A/C (*LMNA*) mutations are a relatively common cause of DCM (6–8% of all idiopathic DCM) and are associated with a high risk for SCD. In fact, a study in the New England Journal of Medicine in 2006 found 8 of 19 (42%) patients who underwent permanent pacing and internal cardioverter and defibrillator (ICD) implantation solely on the basis of *LMNA* gene mutations associated with cardiac conduction defects and normal ventricular function received an appropriate ICD intervention.[17]

### Coronary artery anomalies

In the United States, coronary artery anomalies are the second most common cause of SCD in the young. The most common abnormality associated with SCD is origin of the left main coronary ostia from the right sinus of Valsalva when the coronary artery traverses between the aorta and the pulmonary artery.[18–24] Ischemia has been proposed to occur during exertion when the great vessels increase in size and compress the left main coronary artery. Although anomalous origin of the right coronary artery from the left sinus of Valsalva traversing between the aorta and pulmonary artery is far more common,[24–28] it seems to have a much lower association with SCD than the left coronary artery originating from the right sinus. Coronary artery anomalies are unlikely to be identified without imaging studies unless complaints of early fatigue, angina, or exercise-induced syncope lead to a directed evaluation. Further, coronary anomalies may be difficult to screen for with a routine echocardiogram.

Rarely, acquired premature coronary artery disease can appear in an athlete under age 30. Familial predisposition plus other risk factor prevalence can sometimes lead to coronary events resulting from typical atherosclerosis. Attention to risk factors, such as a strong family history, and to the early symptoms of ischemia, angina and other effort-related symptoms should be pursued in younger athletes as in older athletes.

### Arrhythmic channelopathies and primary arrhythmias

A variety of relatively rare conditions can cause primary arrhythmias in young people [Table 2]. Although there are cases where SCD is the first symptom with these conditions, recurrent syncope often precedes more malignant symptoms. Fortunately, the surface 12-lead ECG is abnormal in most of these conditions. So a minimal evaluation of an ECG and a careful history are indicated for recurrent syncope, and have been advocated as screening tests for athletes by many investigators[29] and several organizations.[29,30]

**Table 2:** Primary arrhythmias and SCD

Arrhythmogenic right ventricular dysplasia
Long QT syndrome
Andersen–Tawil syndrome
Short QT syndrome
Brugada syndrome
Catecholaminergic polymorphic ventricular tachycardia
Wolff–Parkinson–White syndrome
Congenital complete heart block

#### Long QT syndrome

The congenital form of the long QT syndrome (LQTS) is a familial genetic disorder that occurs in about 1 in 2500–3500 individuals.[31] It manifests primarily as ventricular repolarization abnormalities caused by cardiac ion channel mutations. For symptomatic patients, the presenting symptom is usually syncope, which is due to the form of ventricular tachycardia known as “torsades de pointes.” The syncope may occur with specific triggers, such as stress, swimming, and loud auditory stimuli, or it may occur when the child is relatively bradycardic, as during rest or sleep. In most cases, the corrected QT interval is prolonged, but there is considerable overlap with the normal distribution of QT intervals in the healthy population. In fact, 15–25% of patients with LQTS may have a QTc that falls within the “normal range.”[32] This has made clinical diagnosis difficult. In 1993, Peter Schwartz developed a set of diagnostic criteria that combined ECG and clinical criteria into a point system to aid in the diagnosis of LQTS.[33] Family history of LQTS or sudden unexplained cardiac death are important factors in these criteria. However, points are also given for syncope, which occurs in up to 30% of the healthy population, and for QTc >450, which can be found in 2–5% of the healthy population. Further clinical evaluation of the QT interval during exercise or with epinephrine infusion can aid in the diagnosis, as QTc prolongation may become more exaggerated at increased heart rates as the QT interval fails to shorten in some patients. There are a variety of genetic abnormalities of ion channels which have now been identified, and it is estimated that up to 80% of patients with a high index of suspicion for LQTS have a mutation in one of the known LQTS genes.[34,35] The specific phenotype may be predicted from the genetic mutation found and may aid in the assessment of risk for sudden death or response to therapy.[36] Currently, the mainstay of therapy remains beta-blockade, which prevents severe symptoms and sudden death in most cases, but may be less effective for LQTS3. If symptoms recur on beta-blocker therapy, implantation of an ICD is generally considered indicated.[37]

#### Catecholaminergic polymorphic ventricular tachycardia

Catecholaminergic polymorphic ventricular tachycardia (CPVT) is characterized by ventricular ectopy induced by exercise or emotional stress.[38] It is a genetic channelopathy, most commonly caused by mutations of the ryanodine receptor gene (*RYR2*) which encodes a sarcoplasmic calcium ion channel.[39] The onset of CPVT symptoms typically occurs in childhood and adolescence. Exercise stress testing is important for diagnosis, since CPVT cannot be diagnosed on surface ECG. During exercise stress testing, ectopy is enhanced at greater levels of activity and often a “bidirectional” VT with a beat-to-beat 180° rotation of the QRS complex is observed. If left untreated, CPVT is lethal in 30–50% of patients. Although beta-blockers are the recommended therapy, many patients will continue to have arrhythmic symptoms and may need to have an ICD placed.

#### Brugada’s syndrome

Brugada’s syndrome is an inherited arrhythmogenic syndrome characterized by specific ECG abnormalities and life-threatening ventricular arrhythmias.[40] It commonly presents in men in the fourth decade as syncope and ventricular arrhythmias. The characteristic ECG findings are coved or saddle-back ST-segment elevation in leads V1–V3 with complete or incomplete right bundle branch block and T-wave inversion. The ECG abnormalities may not be evident until unmasked by an infusion of a sodium channel blocker (flecainide, ajmaline or procainamide). A handful of genetic mutations have been discovered to cause Brugada’s syndrome; however, *SCN5A* is the most commonly occurring defect. Treatment is limited to ICD implantation.

#### Arrhythmogenic right ventricular dysplasia

Arrhythmogenic right ventricular dysplasia or cardiomyopathy (ARVD/ARVC) is a rare cause of SCD in the United States, but has been reported as the most common cause of SCD in the young athletes in Italy.[41] Clinical presentation typically occurs in early adolescence to young adulthood. It is a heritable, progressive cardiomyopathy characterized by fatty and fibrous replacement of the myocardium, classically causing thinning of the right ventricular (RV) free wall. However, biventricular and left-dominant patterns are now being recognized.[42,43] It is also characterized by electrical instability, and patients may present with a variety of ventricular arrhythmias, which are often provoked by exercise. The diagnosis is often difficult to make clinically. The RV changes are very hard to detect by echocardiography, but if suspicion is high, it can be detected by RV angiography and magnetic resonance imaging (MRI).[44] The resting ECG may be mildly abnormal with ventricular premature beats with a left bundle morphology, T-wave inversion in leads V2 and V3, a so-called epsilon wave following the QRS in lead V1, and a wide QRS in leads V1–V3. Genetic testing may aid in the diagnosis, particularly if there is a strong family history. Although both drug therapy and catheter ablation are occasionally successful, implantation of an ICD is usually recommended for patients with significant symptoms.

### Other causes

#### Commotio cordis

Commotio cordis, defined as SCD due to a relatively innocent chest wall impact in individuals with a normal heart, is a very rare cause of SCD in young people, although it may be underestimated in its frequency.[45,46] Victims are typically males less than 18 years of age. Commotio cordis requires a blow directly over the heart, exquisitely timed to within a narrow 10–30 msec window just before the T-wave peak during the vulnerable phase of repolarization.[47] Collapse may occur immediately or after light-headedness for a period of few seconds. When an initial rhythm is documented, it is usually ventricular fibrillation. Only about 25–35% of commotio cordis victims survive, usually associated with timely cardiopulmonary resuscitation (CPR) and defibrillation. Since most reported cases have occurred in sports such as baseball, it had been thought that prevention could be best accomplished by a combination of chest protectors and reduced impact force baseballs. However, these interventions are proving inadequate for prevention of sudden deaths from commotio cordis in recent studies.[48,49]

#### Congenital heart disease

The incidence of sudden death in patients with congenital heart disease is about 100/100,000 patient years.[50] It is highest in cyanotic and left heart obstructive lesions and may be due to arrhythmic, embolic, or circulatory phenomenon. The risk of sudden death appears to increase with age and time since surgery. Certain congenital defects have a high associated risk of acquired arrhythmias following repair. Specifically, tetralogy of Fallot is associated with higher incidence rates of known ventricular tachycardia and intra-atrial re-entry tachycardia and a 0.5–6% risk of SCD. Patients with both single-ventricle physiology status-post Fontan, and transposition of the great arteries status-post atrial switch (Senning or Mustard) also have high acquired arrhythmia rates with increased incidence of SCD.

## SCREENING FOR SCD RISK IN THE YOUNG

As reviewed above, sports participation has been associated with an increased risk of SCD in young people. Therefore, cardiovascular screening for conditions that could lead to an increased risk of sudden death has focused mainly on the pre-participation screening of athletes. In the United States, current recommendations are for a focused personal and family history of the athlete with special emphasis on history of exertional chest pain, syncope or a family history of early sudden death, as well as examination for blood pressure, murmurs, and stigmata of Marfan’s syndrome.[51] If any abnormalities are found, additional studies are initiated to systematically exclude known causes of sudden death. A relatively intense debate has been ensuing over the effectiveness of universal electrocardiogram (ECG) screening for athletes and/or all infants.[30] For the past 25 years in Italy, all athletes have undergone ECG) screening, with some data indicating fewer deaths during athletic activities associated with institution of the screening program.[29,52] Although the reduction in SCD has been related to disqualification of young people found to have HCM, ARVC and other rare abnormalities, the current reported rate of SCD (about 0.8/100,000 per year)[52] is not very different from that reported for unscreened athletes in the United States.[53] These data and the relatively large number of estimated eligible athletes in the United States that would require screening (10-12 million) have led to the current recommendations against universal ECG screening in the US. The cost of the ECG and its interpretation, in addition to further testing due to frequent false positive results, has been determined to have an unfavorable cost-benefit ratio. However, new guidelines are currently being vetted and the situation may change as more data are available.

## GENERAL TREATMENT AND PREVENTION OF SCD IN THE YOUNG

Although each of the conditions discussed above have specific therapies, the most effective immediate treatment to change SCD into resuscitated SCD is to increase the prevalence of CPR training in the general population, and the availability of automatic external defibrillators (AEDs). AEDs are best located in places where large groups of people gather, including where young athletes may be competing. Such devices have continued to decrease in cost and have improved ease of use, but financial and other barriers still remain to their widespread deployment.

For survivors of near SCD events, systematic evaluation should be performed to determine the cause of the event and treatment should be focused around the underlying etiology. For many of the underlying cardiac pathologies, an ICD is indicated.

## SUMMARY

Sudden death in the young is rare. About 25% of cases occur during sports. Most young people with SCD have underlying heart disease, with HCM and coronary artery anomalies being most common in most series. ARVD and LQTS are the most common primary arrhythmic causes of SCD. It is estimated that early CPR and widespread availability of AEDs could prevent about 25% of pediatric sudden deaths.
